# Death

**Published:** 1887-04-09

**Authors:** 


### WORDS OF CONSOLATION.

[Communications for this column should be addressed, "Chaplain," care of the Editor of THE HOSPITAL, who will gratefully welcome any suggestions or inquiries from matrons, nurses, and the staff generally.]

### XXVIII.—DEATH.

THE first words our blessed Lord uttered when He heard the message, "Thy daughter is dead," delivered to Jairus, were "Fear not" (Luke viii, 50). First applied to the ruler of the synagogue, the words were intended, no doubt, to calm his troubled spirit after many restless hours of watching by the sick-bed, and so to prepare him for the exercise of a greater faith in our Lord's person and power than he had previously shown. When Jairus first sent to Jesus he gave Him credit for being able to heal sickness; but his faith (or at least the faith of his household) scarcely admitted the possibility of life being restored when once the spirit of the maid had departed. Therefore, said the messenger, "Trouble not the Master." Jairus more than feared that the message had been delivered too late. He feared death; he did not fear the sickness. So long as that lasted he felt it would be well if Jesus came. But he did fear the *end*. He could not think of *that* as a sleep, from which his daughter might be awakened. In sickness, in pain, in watching, in all that really does make the last hours of a loved one so distressing, he had not feared, because he had hoped. In ignorance he feared the painless end of all this more than the pain which preceded it. He saw no remedy when the spirit of the child had gone. Why should he trouble the Master? Possibly Jairus himself may not have shared all the gloomy fears of his household. But it is not at all difficult to understand the general change of mind which had supervened in that sorrowful family when they suspected that Jesus had arrived too late. They were weeping, they had lost faith, they feared. Our Lord rebukes each weakness in turn. They were not to weep, they were to believe; but, first of all, they were not to fear. Upon these conditions the truer version of the dread tale of death would be expounded to them, and they would each learn to look forward to their own death-bed (and to others as well), not as an end to be dreaded, and because so much dreaded the less thought about the better, but as a period in life very near in its resemblance to the experience so common of "falling asleep." Again, it matters little whether the interval between the departure of the spirit, and its recall at the voice of Jesus, be a few minutes, as perhaps it was in the case of Jairus's daughter, or a few hours, as it was with the widow's son at Nain, or a few days, as it was with Lazarus, or a few years, as it may be with ourselves—the time is nothing. It is equally true of all who die in Christ; "they are not dead, but sleeping."

"Thou knowest, Lord, the secrets of our hearts (and that in many of them lurks a secret fear of death). Shut not Thy merciful ears to our prayer, but spare us, Lord, most holy. O God, most mighty, O holy and merciful Saviour, Thou most worthy Judge eternal, suffer us not, at our last hour, for any pains of death to fall from Thee."

(*To be continued.*)

